# Community Landscapes: An Integrative Approach to Determine Overlapping Network Module Hierarchy, Identify Key Nodes and Predict Network Dynamics

**DOI:** 10.1371/journal.pone.0012528

**Published:** 2010-09-02

**Authors:** István A. Kovács, Robin Palotai, Máté S. Szalay, Peter Csermely

**Affiliations:** 1 Department of Medical Chemistry, Semmelweis University, Budapest, Hungary; 2 Department of Physics, Loránd Eötvös University, Budapest, Hungary; Indiana University, United States of America

## Abstract

**Background:**

Network communities help the functional organization and evolution of complex networks. However, the development of a method, which is both fast and accurate, provides modular overlaps and partitions of a heterogeneous network, has proven to be rather difficult.

**Methodology/Principal Findings:**

Here we introduce the novel concept of ModuLand, an integrative method family determining overlapping network modules as hills of an influence function-based, centrality-type community landscape, and including several widely used modularization methods as special cases. As various adaptations of the method family, we developed several algorithms, which provide an efficient analysis of weighted and directed networks, and (1) determine pervasively overlapping modules with high resolution; (2) uncover a detailed hierarchical network structure allowing an efficient, zoom-in analysis of large networks; (3) allow the determination of key network nodes and (4) help to predict network dynamics.

**Conclusions/Significance:**

The concept opens a wide range of possibilities to develop new approaches and applications including network routing, classification, comparison and prediction.

## Introduction

In real networks, module or community structure plays a central role in the understanding of topology and dynamics. Numerous module determination methods are based on the intuitive picture identifying the network communities as dense groups of the network, whose nodes have a much stronger influence on each other than on the rest of the network. The development of a method, which translates this intuitive definition of modules into a practically applicable, fast, accurate and widely usable algorithm turned out to be a very challenging problem. So far a wide variety of great ideas and powerful approaches based on very different physical or algorithmic grounds were applied in order to solve this problem. At the moment there is no ‘best method’ available to find network modules, and even the widely used algorithms may suffer from serious problems (see Figure S1.1, and Tables S1.1 and S1.2 in the Electronic Supplementary Material S1) [1]–[7], although they usually provide useful and clear dissections of networks.

In 2002 Girvan and Newman published a seminal paper [2] using an algorithm for detecting communities by iteratively removing edges of high betweenness centrality values from the network, and defining communities as the connected components of the network after these edge removals. Later they [8] introduced the modularity measure, Q with which the optimal number of removed edges could be determined. In a short time the Q function evaluating the goodness of partitioning a graph into given clusters became an essential element of a wide range of clustering methods. Different kind of approaches have also been developed, including ones utilizing spectral functions of the graphs [9], [10], dynamic algorithms like random walks [5], [11], spin models (e.g. the Potts model) [12] or synchronization models [13]. The most popular method to find overlapping communities is the Clique Percolation Method described by Palla et al. in [7], but other excellent methods optimizing overlapping quality functions such as that of Nepusz et al. [4] or the link-based method resulting in pervasive overlaps [14] also exist. Although the field of community detection is quite diversified, we tried to collect the main algorithms in Table S1.2 in the Electronic Supplementary Material S1, and we also recommend a current extensive review of the field by Santo Fortunato [1].

In this paper we introduce an integrative network module determination method family, called ModuLand (see Box 1 for the glossary of novel terms). This module determination method family is based on the novel concept of understanding the overlapping modules as hills of an influence function-based, centrality-type community landscape. The ModuLand method family gives a common framework for the development and comparison of a large variety of modularization methods resulting in network modules with variable overlaps, requiring different computational speed and providing a different level of accuracy.

**Box 1. Glossary. Here we present a short guide to the algorithms and methods defined in this paper and in the Electronic Supplementary Material S1 in detail.**

**GradientHill method**: a local maxima-based module determination approach, in which the module membership value of a link is determined by the module membership value(s) only of its neighboring link(s) having maximal centrality values (see Section V.2.b. in the Electronic Supplementary Material S1).

**LinkLand algorithm**: an influence function calculation method starting from a given link in undirected networks (see main text and Section IV.1.b. in the Electronic Supplementary Material S1).

**ModuLand method family**: the integrative name of our module determination approach based on the hills of the community landscape (see main text and Section II. in the Electronic Supplementary Material S1).

**NodeLand algorithm**: an influence function calculation method starting from a given node in undirected networks (see main text and Section IV.1.a. in the Electronic Supplementary Material S1).

**PerturLand algorithm**: an influence function calculation method starting from a given node in directed networks (see Section IV.2. in the Electronic Supplementary Material S1).

**ProportionalHill method**: a local maxima-based module determination approach, in which the module membership value of a link is determined by the module membership values only of its neighboring links having non lower centrality values (see main text and Section V.2.b. in the Electronic Supplementary Material S1).

**TotalHill method**: a local maxima-based module determination approach, in which the module membership value of a link is determined by all the module membership values of its neighboring links (see Sections V.2.c. and V.2.d. in the Electronic Supplementary Material S1).

## Results

### Description of the ModuLand Method Family

**Figure 1:**
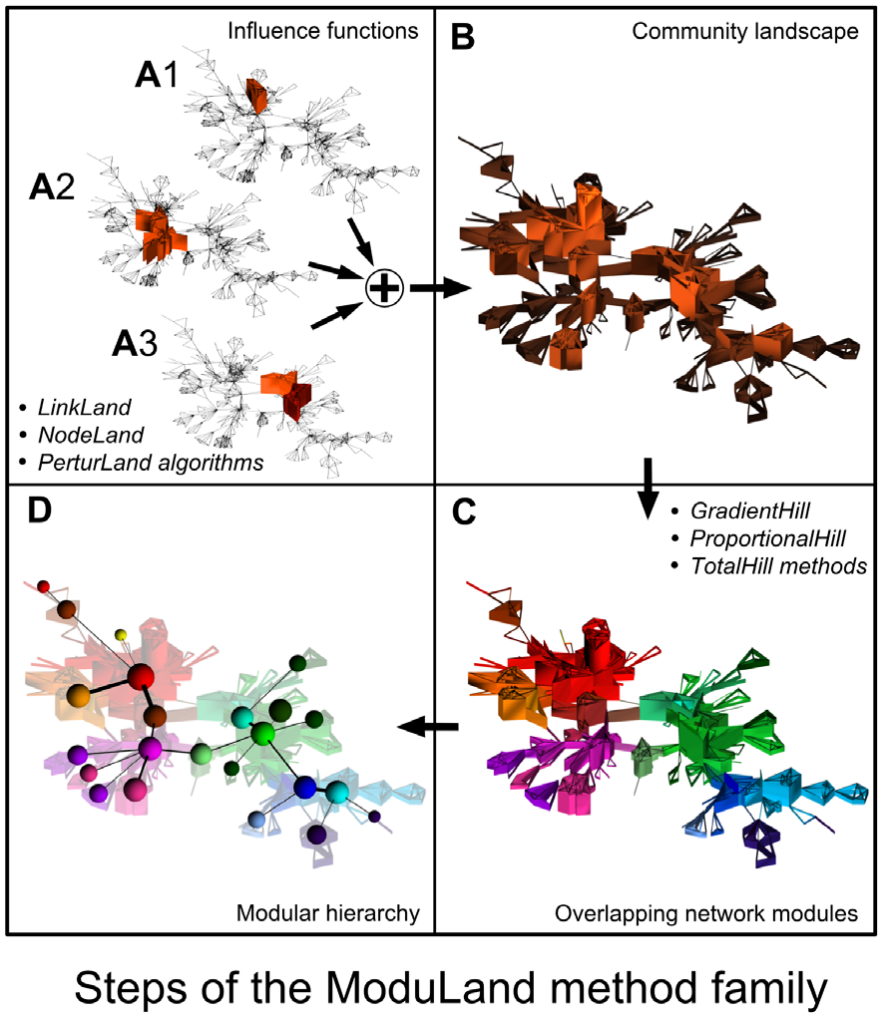
Description of the ModuLand method family. For this illustrative example we used the network science co-authorship network [15] without link weights using the LinkLand influence function calculation method with the TotalHill module membership assignment method. The network was laid out using the Kamada-Kawai algorithm and was visualized with a custom Blender script. On the vertical axes influence function values (panel A), or community landscape values (panels B, C and D) of the links are shown. Influence functions of panels A1, or A2 belong to the Barabási-Vicsek, or Girvan-Newman author-pairs, respectively. Panel A3 shows the merged influence function of the Arenas-Pastor-Satorras and Guimera-Amaral co-authorship links. Links and nodes of panels C and D are colored in proportion of the colors of the modules they belong. Panel A: influence function calculation. First, the influence function of each link (or node) of the network were identified. If a link is in the ‘middle’ of a module, it is affected by many influence functions (all the three widely collaborating author-pairs, whose influence functions are shown by the arrows, are from this category). On the contrary, links at module ‘edges’ are affected by few influence functions only. At the bottom of the panel the names of the three algorithms we described in details are shown. Panel B: community landscape construction. Next, the community landscape is constructed by summing up the influence function values for all nodes or links. The hills of the community landscape correspond to the modules of the network. Panel C: determination of overlapping modules. Last, modular centers are identified as the links at the local maxima of the community landscapes, and memberships of links in all network modules are determined. At the top of the panel the names of the three methods we described in details are shown. Panel D: determination of network hierarchy. Optionally, a higher level hierarchical representation of the network can be created, where nodes of the higher level correspond to modules of the original network, and links of the higher level correspond to overlaps between the respective modules. Sizes of higher level nodes correspond to the log size of the respective lower level modules, where the module size is the sum of the membership assignment strengths of all nodes to that module.

Keeping in mind the emerging needs for an integrative approach for the determination of network modules, we have developed the ModuLand method family (Figure 1 and Figure S1.2 in the Electronic Supplementary Material S1). All members of the ModuLand method family are based on the following common steps:

1. Determination of influence functions: If a node lies in a module, than its influence on the links of the given module is typically larger than on more distant links of the network. As a first step, we determine the influence function, *f_i_* of each node *i* of the network on the links of the entire network. After perturbation-flow type calculations detailed below starting from each node *i* of the network, we acquire a set of 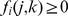 values over all links *(j,k)* of the network for node *i*.
2. Construction of a community landscape: The influence functions of different nodes in the same module are generally different. Nevertheless, the module is the set of nodes, which mutually have a large influence on each other. In order to take this mutuality into account, we summarize the influence function values of Step 1 over each link of the network: 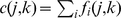. The resulting *c(j,k)* values represent a smooth, centrality-type quantity, which is larger for the module cores and smaller for the surrounding regions. We represent these non-negative *c* values as a vertical measure forming a community landscape over the links of the 2D visualization of the original network (Figure S1.3 in the Electronic Supplementary Material S1).
3. Determination of hills of the community landscape: Modules are determined as the ‘mountains and hills’ of the community landscape of Step 2. We present two different approaches:
  - Modules are the connected components above a chosen centrality-threshold.
  - Modules are determined by local maxima of the community landscape and their surrounding region (Figures S1.4 and S1.5 in the Electronic Supplementary Material S1).
4. Determination of a hierarchy of higher level networks: We note that a higher level network of the modules of Step 3 can also be constructed, where each former module is a node of this higher level network. If the higher level networks are re-assessed with the ModuLand method again and again, a set of hierarchical layers of modules can be defined until the giant component of the whole network coalesces to a single node (Figure S1.6 in the Electronic Supplementary Material S1).

In the followings we will describe the four major steps of the ModuLand method in detail.

#### Step 1: Determination of influence functions

In principle, the determination of the influence functions (or indirect impact of a node or link) requires a network-dependent perturbation-flow simulation on the network (as an example, see our PerturLand algorithm in Section IV.2. in the Electronic Supplementary Material S1), which is a challenging problem in itself. However, the details of the influence functions usually average out during the community landscape construction, which justifies the use of less specific, faster approximations. Here we present our simplest influence function calculation algorithm, the NodeLand algorithm in detail, which can be applied on weighted, undirected networks.

NodeLand algorithm: starting from a given node *s*, the NodeLand algorithm iteratively determines the set of nodes *A*, which is strongly influenced by node *s*. For any given *A* set, we define the density of the set as
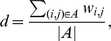
where *w_ij_* is the weight of the link between node *i* and *j*, and *|A|* is the number of nodes in *A*. Initially, *A* consists of the staring node only, thus 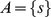. In each iterative step we will expand *A*. For each neighboring node 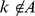 we determine the potential new density value, including node *k* in *A*:
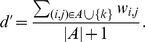
If the density of *A* can be increased this way, then we add the nodes with maximal 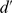 value to the set *A*, and start a new step of the iteration. We stop the process, when the density can not be further increased with the addition of single neighboring nodes. At this point, we will have a final set *A* containing the nodes strongly influenced by the starting node, *s* (including *s* itself). The influence function *f_s_* over the links is defined as 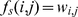, if 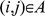 and zero otherwise.

LinkLand algorithm: the LinkLand algorithm, used in our module determinations of the main text below, differs from the NodeLand algorithm in two points.

- In the LinkLand algorithm the influence functions are assigned to starting links instead of starting nodes, thus initially *A = {k,l}* containing the two end-nodes of the starting link (*k,l*).
- In contrast to the NodeLand algorithm, while calculating the influence function the weight of the starting link (*k,l*) is also taken into account: 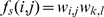, if 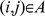, and zero otherwise.

On undirected networks we prefer to use the LinkLand algorithm, which is found to provide an acceptable compromise between precision and speed. Identification of the influence function of a node or link in the case of NodeLand and LinkLand algorithms is structurally similar to a breadth-first search, therefore the worst-case runtime complexity of the two algorithms for all nodes or links is *O(n(n+e))* and *O(e(n+e))*, respectively, where *n* is the number of nodes and *e* is the number of links in the network. However, in practice these algorithms are very fast as the influence zone of any given starting node or link rarely covers the whole network. For downloading the ModuLand program package including the NodeLand, LinkLand influence function calculation algorithms and their User Guide, see our homepage: http://www.linkgroup.hu/modules.php.

As an example for the results obtained by the LinkLand algorithm, Figure 1A shows three influence functions defined over the links of the ‘network science’ co-authorship network [15]. All the three starting links highlighted by the arrows belong to widely collaborating, key players of the field, resulting extended influence zones.

#### Step 2: Construction of a community landscape

In order to find the regions with nodes mutually having a relatively large influence on each other, we calculate the sum of the individual influence functions on a given network link resulting in the 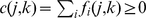 centrality-type value. From the centrality of the links the centrality of the nodes can be derived by a summation: 
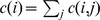
. When mentioning ‘centrality’, throughout the paper we refer to these definitions. The centrality values can be plotted on a vertical scale resulting in a community landscape over a 2D representation of the links of the network (see Figure 1B and Figure S1.3 in the Electronic Supplementary Material S1). Now we can see the ‘hills and mountains’ of the community landscape, consisting of the nodes influencing each other stronger than the rest of the network. This is exactly the intuitive definition of modules given in the first section of this paper. The precise definition of these hills will be the subject of the following section.

#### Step 3: Determination of hills of the community landscape

Here we present two main approaches of hill-determination suitable for the determination of modules.

Centrality threshold based hills: as a natural choice, hills may be identified as the connected components of the community landscape above a given threshold. This approach results in distinct network modules without overlaps, like in case of the widely used Girvan and Newman method [2]. Generally, it is a rather difficult problem to choose the most appropriate value for the threshold (see Figure S1.4 and Table S1.2 in the Electronic Supplementary Material S1). On one hand, if we raise the detection limit too high, we will find only the largest communities. On the other hand, if we set the detection limit too low in order to be able to see the smaller modules, then most of the large communities would merge together. This is the manifestation of the well known giant-component problem [6], [16]–[18]. As the centrality threshold-based approach is very general, most of the former methods yielding non-overlapping modules can be interpreted in the ModuLand method family as the application of the threshold-based hill determination method over an appropriate community landscape.

Local maxima based hills: in this method we start the identification of the modules by finding the module centers, which are identical with the hill-tops or local maxima of the community landscape, defined as follows:

- Undirected networks: A hill-top of the community landscape contains all connected links having the same, locally maximal centrality value, while having all of their neighboring links with lower centrality values.
- Directed networks: The definition of hill-tops is more complicated in directed networks, but we also show it here for clarity. Let the outbound links of a link *(i,j)* denote the outbound links of its end-point, node *j*. Then a hill-top is either a single link with all of its outbound links having a lower centrality, or a strongly connected component (meaning that every node of the component is reachable from every other node by a directed walk on this component) consisting of all links of the same centrality with all of their outbound links having lower centralities.

By this definition the number of local maxima automatically yields the number of modules, and at the same time all small and large modules are identified simultaneously. This is in strong contrast to the previously described threshold-based approach, which often needs special criteria to determine the threshold value.

At this stage only the central links or plateaus of the modules have been identified. In the next step, the modules will be extended towards lower regions of the community landscape. We have developed several methods for this extension process detailed in Section V.2. in the Electronic Supplementary Material S1. We suggest the use of the ProportionalHill method described below, determining the module-membership values of the links proportionally to the membership values of their neighbors located higher in the community landscape. Using this method the hills will naturally overlap, resulting in links which are assigned to multiple modules simultaneously.

ProportionalHill method for the determination of network modules: here we present the algorithm of the ProportionalHill method for undirected networks, while the analogous directed version can be found in Section V.2.b. in the Electronic Supplementary Material S1. As the first step, the community landscape is divided into hills (corresponding to network modules) defined by the local maxima of the community landscape described above. For a given link (*i,j*), 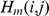 is the hill (or module) membership value of the link in the *m*-th hill. The module membership values are normalized to the centrality of the given link, 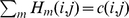. If link *(i,j)* is part of the *module center* of module *m*, then let 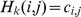, if 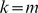, and 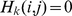 otherwise. For the other links we apply the following rule:
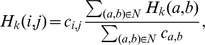
where *N* is the set of neighboring links of link *(i,j)* having larger or equal centrality than that of link *(i,j)*. Now the community landscape is divided into multiple hills identified as the modules of the network. Thus, 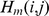 readily gives the module membership value of link *(i,j)* in module *m*. Finally, the module membership values of a given node *i* is given by 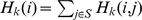, where *S* is the set of the neighboring nodes of node *i* and *k* is the module considered. The presented ProportionalHill method has a runtime complexity of *O(edM)*, with *e* being the number of links, *d* being the average node degree and *M* being the number of the identified modules.

If we need smaller or larger overlaps between the modules, than those obtained with the ProportionalHill method, we may use the GradientHill or TotalHill methods, respectively, as described in Section V.2. in the Electronic Supplementary Material S1 and Figure S1.5 in the Electronic Supplementary Material S1. While for practical purposes we suggest the use the ProportionalHill method, the most detailed module overlap information is acquirable with the computationally more expensive TotalHill method. The TotalHill method also takes into account the neighboring links of lower centrality during the module-extension step. The TotalHill approach requires the solution of *M* appropriate linear equation systems of size *n* by *n*, with *n* being the number of nodes. Results obtained using the TotalHill method can be seen on Figure 1C and Figure S1.3 in the Electronic Supplementary Material S1, where large segments of the network belong to at least two modules. For downloading the ModuLand program package including the ProportionalHill and TotalHill method algorithms and their User Guide, see our homepage: http://www.linkgroup.hu/modules.php.

#### Step 4: Determination of a hierarchy of higher level networks

Optionally, a higher level hierarchical representation of the network can also be created, where the nodes of the higher level correspond to the modules of the original network, and the links of the higher level correspond to the overlaps between the respective modules (Figure 1D, Figures S1.2 and S1.6 in the Electronic Supplementary Material S1).

In the description of the calculation of the higher hierarchical level let us consider here the undirected case only (the directed case is described in Section VII. in the Electronic Supplementary Material S1). Let the strength of the overlap (equaling with the weight of the link at the one level higher hierarchy) between modules *i* and *j* be the sum of the node-wise calculated overlap values *O_ij_(n)*:
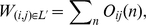
where *O_ij_(n)* is proportional to the module membership values *H_i_(n)* and *H_j_(n)* and being normalized to the centrality as:
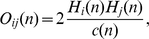
where 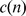 is the centrality of node *n*, and the factor of *2* refers to that both directions between the modules have been taken into account.

The steps leading to a higher level hierarchical representation can be applied repetitively until the giant component of the whole original network is represented by a single node allowing a fast, zoom-in type analysis of large networks (Section VII. in the Electronic Supplementary Material S1).

A simple case illustrating this scenario can be seen on Figure S1.6 in the Electronic Supplementary Material S1 showing the hierarchical levels of the network science collaboration network [15]. It can be seen that the modules of higher and higher hierarchical levels correspond to larger and larger groups (e.g. the modules of the modules etc.) of the original network nodes.

### Characterization of the Overlapping Modules Identified by the ModuLand Method Family

The ModuLand method family, even with its simplest NodeLand influence function calculation method correctly identified the observed split of the gold-standard Zachary karate club network [19], while uncovering a third, previously identified module and several club-members in modular overlaps (Figure S1.7 in the Electronic Supplementary Material S1).

Application of the LinkLand influence function calculation method to the University of South Florida word association network [20] resulted in a set of modules having a highly heterogeneous degree, module size and module overlap distribution (Figure S1.8 in the Electronic Supplementary Material S1), which is in agreement with earlier data (see Supplementary Discussion in the Electronic Supplementary Material S1) [3], [7].

The application of the ModuLand method on the benchmark graphs of Lancichinetti et al. [21] generated over a range of parameter settings showed (Figure S1.13. and Section VI.2. in the Electronic Supplementary Material S1) that the identified ModuLand modules corresponded consistently to the original modules, while modules can be defined in the strong sense (where ‘strong sense’ means, at least the half of the neighboring nodes are assigned to the same module as the given node, see ref. [21] and Table S1.3 in the Electronic Supplementary Material S1).

**Figure 2:**
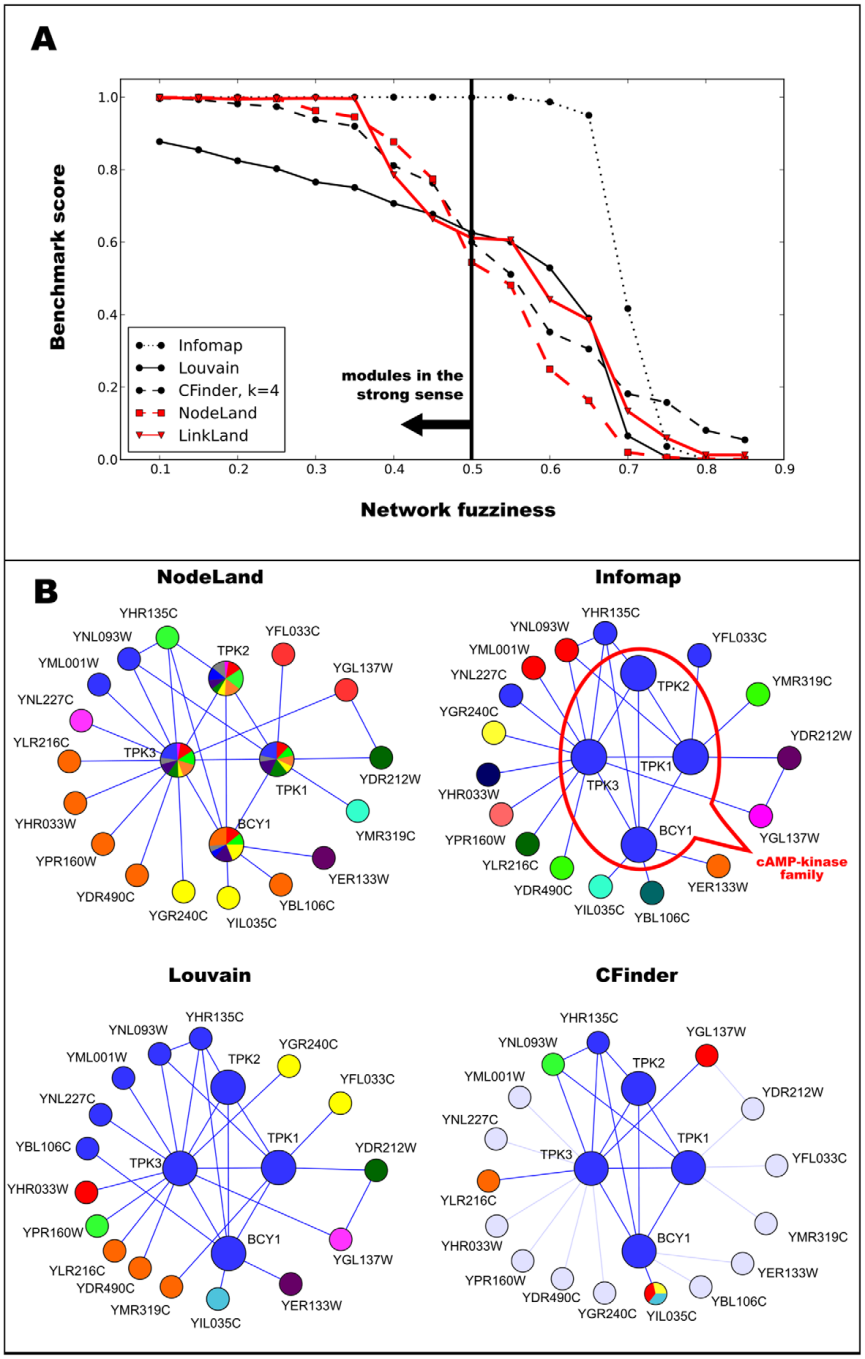
Comparison of the ModuLand method with other modularization methods. Panel A: Comparison of the identified modules with the modules of the benchmark graph of Lancichinetti et al. [21]. Modularization has been performed on benchmark graphs with degree and module size distribution exponents γ = 2 and β = 1 using the NodeLand or LinkLand influence function calculation algorithm with the ProportionalHill module membership assignment method with merging highly correlated modules using an arbitrary chosen correlation threshold of 0.9 (see Section VI.1. in the Electronic Supplementary Material S1; red squares with dashed line and red rectangles with solid line for NodeLand and LinkLand, respectively), the InfoMap method (black circles with dotted line, [5]), the Louvain method (black circles with solid line, [22]) and the CFinder method with k = 4 cliques (black circles with dashed line, [7]). The number of nodes of the benchmark graphs was N = 1000, the maximum degree was K_max_ = 50, the average degree was K = 15 and the network fuzziness μ of the x-axis of Panel A) was ranging from 0.1 to 0.85, where μ>0.5 means that the modules are no longer defined in the strong sense. Higher normalized mutual information (shown on the y-axis) represents a better recovery of the original modules. The panel shows the averaged results of 50 representations. Panel B: comparison of module assignment of the cAMP-dependent protein kinase family in the yeast protein-protein interaction network. The panel shows the modular assignment of the 3 catalytic and the regulatory subunit of the yeast cAMP-dependent protein kinase together with that of their first neighbors in the high fidelity protein-protein interaction network of Ekman et al. [25]. For the sake of simplicity only intra-subnetwork contacts have been included. The top left, top right, bottom left and bottom right figures show the modular assignment using the NodeLand, InfoMap, Louvain and CFinder methods, respectively, determined as described in the legend of Panel A. Various colors correspond to different modules. Overlapping ModuLand modules of cAMP kinase members and CFinder modules of their casein kinase II neighbor are marked with pie-charts, where the area of color-codes is proportional to the module membership value of the given node in the given module. To simplify the figure, in case of the NodeLand method (top right figure of Panel B) all neighbors of cAMP-dependent protein kinase family members (which should all have similar pie-charts to the 4 central members due to their multiple modules) were assigned to their maximal modules only.

To obtain a more detailed picture we directly compared the method-pair of NodeLand, or LinkLand influence function calculation algorithm and the ProportionalHill hillfinder method with the InfoMap method [5] Louvain method [22] and the CFinder method using k = 4 cliques [7]. Figure 2A shows the accuracy of the 4 methods in terms of generalized normalized mutual information [23] on the non-overlapping benchmark graphs of Lancichinetti et al. [21]. Our method compares well to the other 3 methods, however, at less defined modules it is not as accurate as the InfoMap method. It is worth to note, that the benchmark graphs used [21] have been developed for the comparison of not overlapping modularization methods, which may – in part – explain why the two overlapping methods did not perform extremely well on them.

Benchmark graphs have been criticized recently due to their limited capacity to reflect the complexity of real-world networks [24]. Therefore, we decided to compare the accuracy of the 4 methods mentioned before on a high-fidelity yeast protein-protein interaction network [25]. The analysis of the modular distribution of a central regulator of yeast cells, the cAMP-dependent kinase family [26] and their neighbors is shown on Figure 2B. While the NodeLand method identified 10 highly overlapping modules (and functions) of the 3 catalytic subunits and their regulatory subunit, the InfoMap, Louvain and CFinder methods all assigned these family members to a single module. These single modules were signaling modules in case of the InfoMap and CFinder methods, while the Louvain method assigned the whole cAMP-dependent kinase family to a vesicular traffic module, which reflects only a small part of their biological regulatory function (Fig 2B and Table S1.4 in the Electronic Supplementary Material S1). The modular assignment of the 16 neighbors of the cAMP-dependent kinase family enriched the modular composition by 4, 11, 8, and 6 modules in case of the NodeLand, InfoMap, Louvain and CFinder methods, respectively. In contrast to the other 3 methods, which assigned all 16 neighbors to various modules, the CFinder found modules for 5 neighbors only (Fig 2B and Table S1.4 in the Electronic Supplementary Material S1). The overlap of the neighbor-enriched cAMP kinase-related modules had similar values between all methods tested (25% to 75% and 33% to 50% agreement between the NodeLand and the other 3 methods vs. in between the other three methods, respectively; Table S1.5 in the Electronic Supplementary Material S1). Similarly, the number of modules, whose functional association with the cAMP kinase family was supported by experimental data, was also in the same range (71%, 58%, 89% and 57% for NodeLand, Infomap, Louvain and CFinder methods, respectively; Table S1.5 in the Electronic Supplementary Material S1).

In conclusion, both i.) the comparison of ModuLand-derived modules with those obtained by other methods and ii.) the experimental data of the literature showed that the pervasive overlaps of the ModuLand method give an adequate representation of the functional multiplicity of protein-protein interaction network nodes. It is important to note that, in contrast to the other methods tested, the ModuLand method gives this rich background of functional information at the single node level as opposed to the subnetwork level of other methods. Moreover, the Moduland-based, different modular assignment strengths of related nodes (such as those of the 3 cAMP-kinase catalytic subunits; Table S1.4 in the Electronic Supplementary Material S1) give a detailed suggestion on the nodes' functional specificity.

### Variable Overlaps of Modules Surrounding Heteronym and Antagonym Words in a Word Association Network

**Figure 3:**
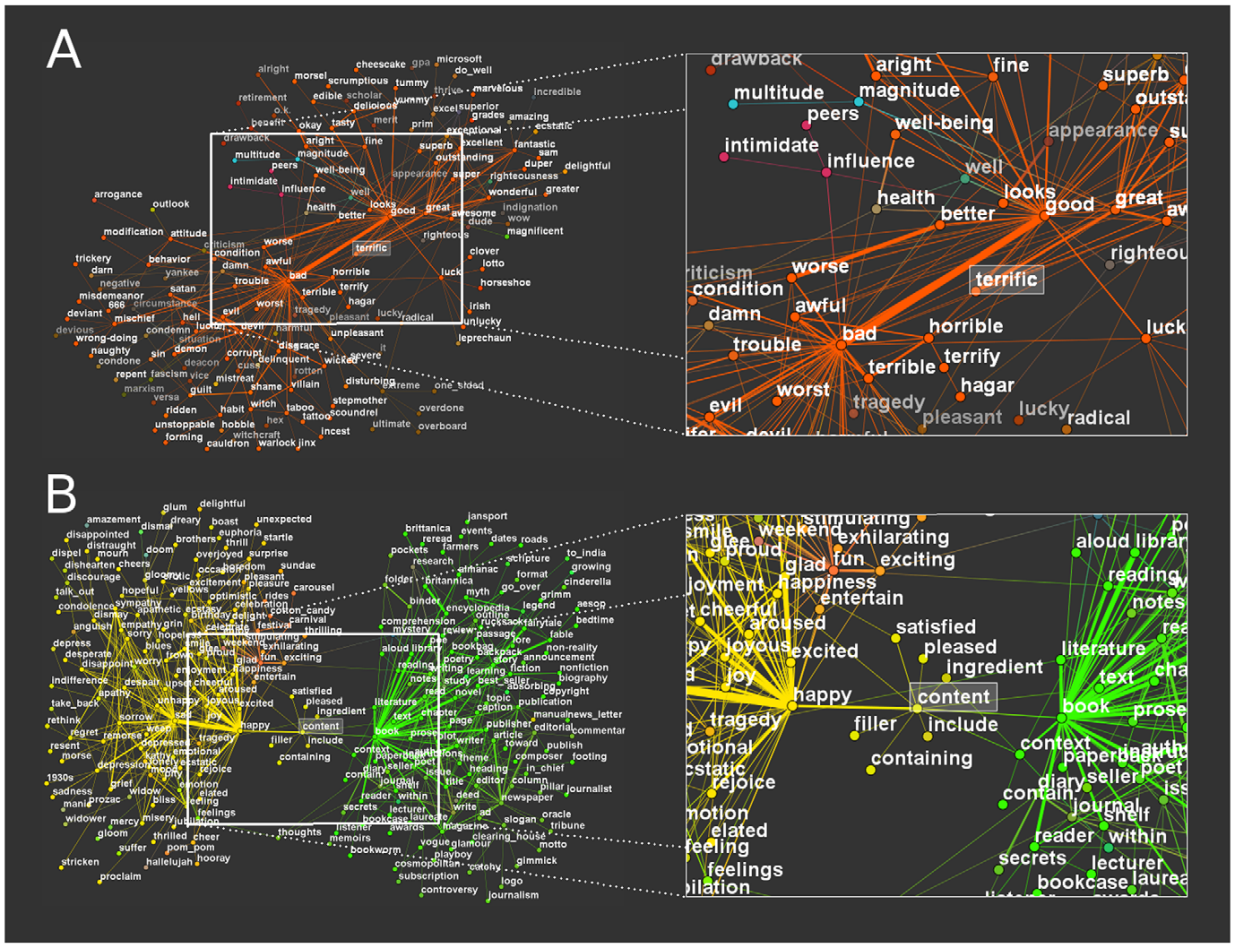
Overlapping modules of a word-association network. Modules of the University of South Florida word association network [20] were determined using the LinkLand influence function calculation method and the TotalHill module membership assignment method. During the post-processing of the module assignment, we merged the modules with ProportionalHill module membership assignment-based correlation higher than 0.9 (see Section VI. in the Electronic Supplementary Material S1, we received similar results without this merging process; data not shown). The network was laid out using the Kamada-Kawai algorithm of Graphviz [48] and visualized using a custom program written in Python language using OpenGL graphics. Links were colored in proportion to the colors of the modules they belong. Panel A: modules around the antagonym word, “terrific”. Panel B: modules around the heteronym word, “content”. In addition to the selected words “terrific” and “content” similar words above a similarity threshold of 10% are also shown with a contrast corresponding to their degree of similarity. The extent of similarity between two words was calculated as the sum of the two pair-wise minima of their unity-normalized module membership vector giving the membership assignment strength of the given word to all modules of the network (for more details see Section V.6.e. in the Electronic Supplementary Material S1).

Extending the analysis of the gold-standard Zachary karate club network, we examined the much larger University of South Florida word association network having 10,617 nodes and 63,788 links [20], which was a target of a successful previous modularization study yielding overlapping modules [7]. This detailed analysis took 10 minutes on a computer with a 3 GHz Intel CPU. Figure 3 shows the modular environment of the antagonym word, “terrific” and that of the heteronym, “content”. The mingling colors indicate a high overlap between the modules. Importantly, the overlap of the modules with alternative meanings of the two words is much greater in the case of “terrific” than in case of “content”, which is a reasonable consequence of the fact that variations of antagonistic meanings (“terrific”) are often amongst our associations, leading to a large joint module containing words with both positive (like “good”, “better” and “great”) and negative (like “bad”, “awful” and “worse”) meanings. We note that the word “well” has multiple meanings, and therefore it is also the member of other distant modules (like the module of “water” or the module connected to “health”). On the contrary to the word “terrific”, the associations between differently pronounced meanings (“content”) are much more seldom. Overlap between the multiple meanings of the words “bright” and “focus” (Figure S1.9 in the Electronic Supplementary Material S1) is closer to that of “terrific” than that of “content”. However, in case of these latter, multiple meaning words the similarly pronounced meanings are not divided into two major sections as in case of the heteronyms, which is again in agreement with our common knowledge.

### Modular Hierarchy of a Social Network

**Figure 4:**
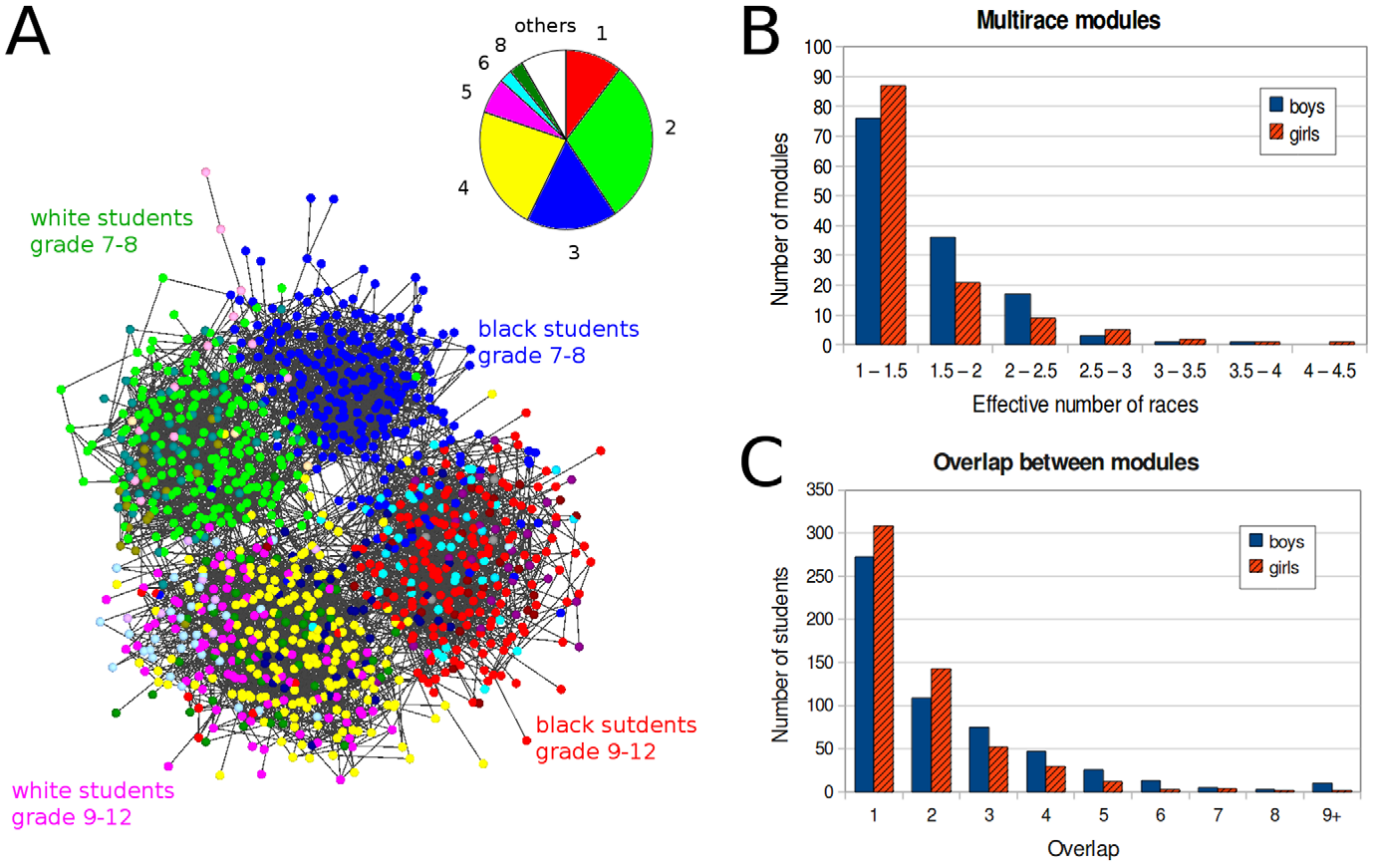
Overlapping modules of a school-friendship network. We have determined the modular structure of Community-44 of the Add Health survey [27] using the LinkLand influence function calculation method together with the ProportionalHill module membership assignment method. During the post-processing of the module assignment, we merged the modules with ProportionalHill module membership assignment-based correlation higher than 0.9 (see Section VI. in the Electronic Supplementary Material S1, we received similar results without this merging process; data not shown). Panel A: modules of Community-44. The school friendship network was laid out using the Kamada-Kawai algorithm. Nodes represent the individual students, and were colored according to the color of the friendship module they assigned the most. We show the modular structure of the first hierarchical level having 18 modules. The inset of Panel A shows color-codes of the modules with an area proportional to the size of the respective module. Panel B: the number of network modules in case of boys (blue, solid bars) and girls (red-black hatched bars) with mixed racial contents at the lowest hierarchical level (level 0). The extent of mixed racial content was monitored using the “effective number of races” (Section V.6.b. in the Electronic Supplementary Material S1) with a bin-size of 0.5. Panel C: overlaps of boys and girls in friendship circles. The number of boys (blue, solid bars) and girls (red-black hatched bars) having different overlaps in friendship circles were determined in the first hierarchical level with a bin-size of 1. Overlap was measured as the “effective number” (Section V.6.b. in the Electronic Supplementary Material S1) of modules of the given student.

The modular hierarchy of the high school friendship Community-44 of the Add-Health dataset [27] was uncovered using several influence function calculation methods. All of these methods revealed four well-distinguishable main modules with a large amount of further sub-modules (Figure 4A and Figures S1.10, S1.11 and S1.12 in the Electronic Supplementary Material S1). Girls were less likely to form multiracial friendship communities (chi-square p<0.05; Figure 4B), and boys were in the overlap of significantly more friendship communities than girls (chi-square p<0.0001; Figure 4C). These differences are in agreement with the sociological observations indicating a larger cohesiveness of friendship circles of girls than that of boys [28], [29].

### Efficient Determination of Central, Key Nodes of Power-Grid Network

**Figure 5:**
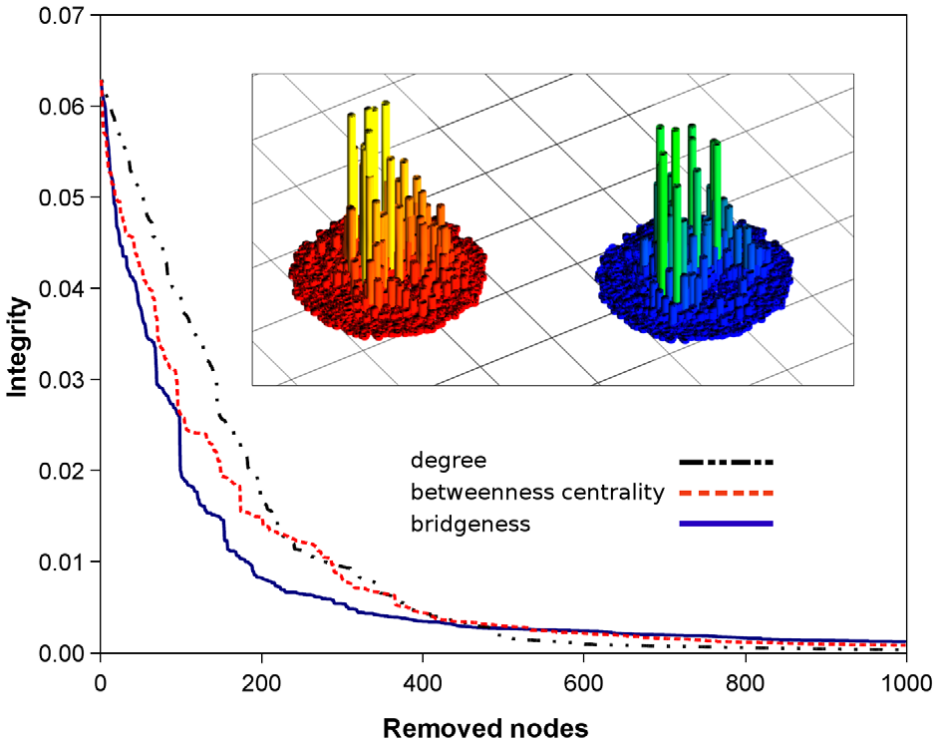
Determination of key nodes of the USA Western Power Grid network. The figure shows the decreasing integrity of the USA Western Power Grid network [31] as a function of the number of nodes removed. Nodes were removed in the order of their decreasing degree (black alternating dashes and dots) betweenness centrality [2] (red dashed lines) or “bridgeness” (solid blue lines), where “bridgeness” measures the overlap of the given node between different modules as described in detail in Section V.6.d. in the Electronic Supplementary Material S1. Network integrity has been calculated after Latora and Marchiori [30]. Bridgeness was calculated from the modular structure of the lowest hierarchical level as determined by the LinkLand influence function calculation method and the TotalHill module membership assignment method. During the post-processing of the module assignment, we merged the modules with ProportionalHill module membership assignment-based correlation higher than 0.9 (see Section VI. in the Electronic Supplementary Material S1, we received similar results without this merging process; data not shown). On the vertical axis of the insets the betweenness centrality (left, color-coded from red to yellow) and bridgeness (right, color-coded from blue to green) of the nodes of the USA Western Power Grid network are shown. Networks on the insets were laid out using the Kamada-Kawai algorithm and visualized with a custom Blender script.

To test whether the ModuLand method family can identify key network nodes, we calculated the change of network integrity [30] during the disintegration of the USA Western Power Grid network [31]. Nodes were removed in the decreasing order of their degree, betweenness centrality and ModuLand bridgeness (measuring the bridge-like role of the nodes between the modules as defined in Section V.6.d. in the Electronic Supplementary Material S1). Figure 5 shows that the impact of bridgeness-based node removal on network integrity was larger than that of the degree-based attacks and was well comparable to, or better than the result of betweenness centrality-based node removal. The equal-to-better performance of bridgeness-based disintegration compared to that using betweenness centrality is surprising all the more, since the global network integrity measure corresponds extremely well to the global betweenness centrality measure [30].

### Discrimination Between Date- and Party-Hubs

**Figure 6:**
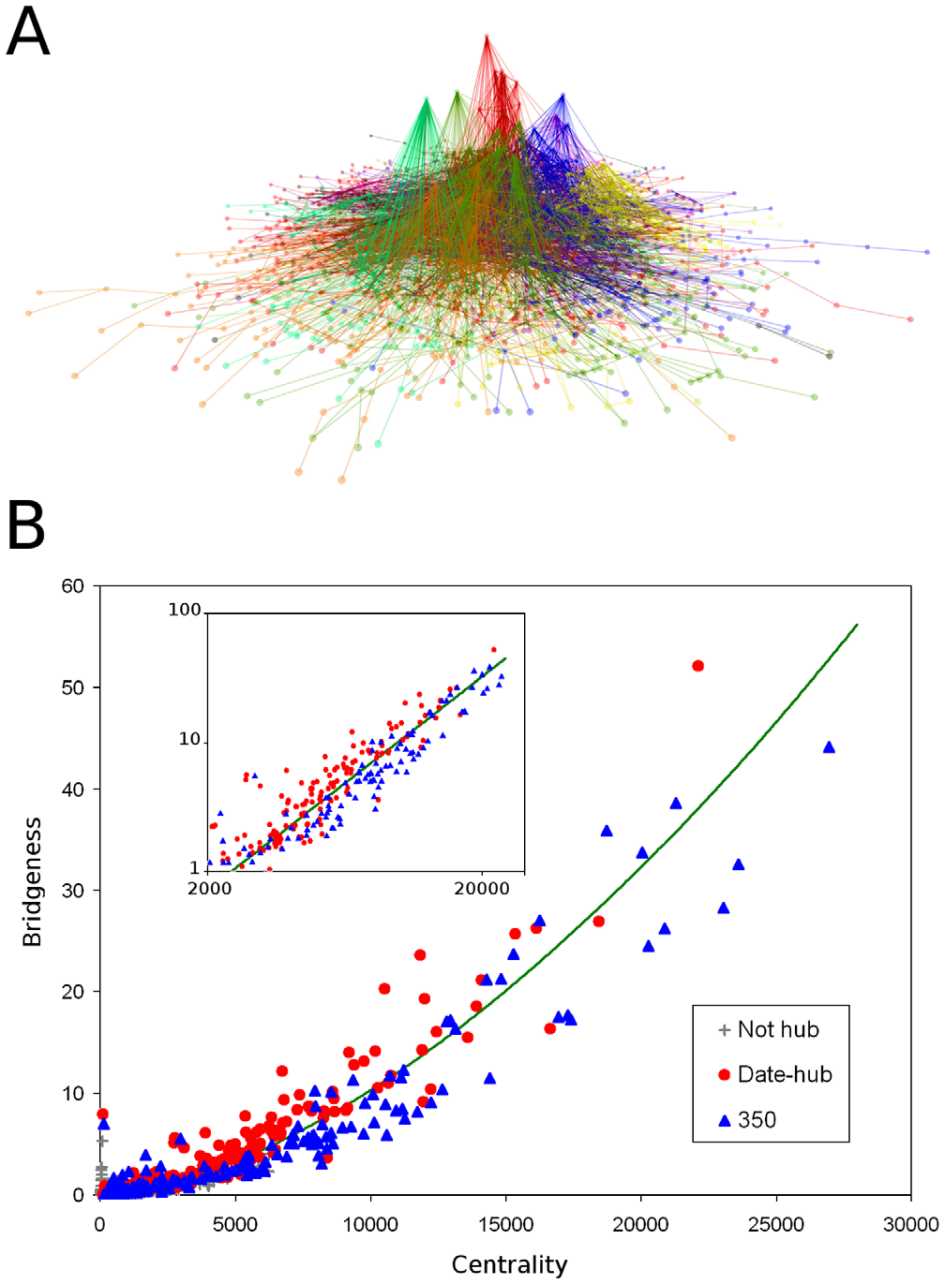
Prediction of the dynamical behavior of network nodes: segregation of date- and party-hubs based on their modular overlaps. Overlapping modules of the yeast protein-protein interaction network of Ekman et al. [25] were identified using the LinkLand influence function calculation method with the TotalHill module membership assignment method using the modular structure of the lowest level of hierarchy. During the post-processing of the module assignment, we merged the modules with ProportionalHill module membership assignment-based correlation higher than 0.9 (see Section VI. in the Electronic Supplementary Material S1, we received similar results without this merging process; data not shown). Panel A: 3D view of the yeast protein-protein interaction network. The underlying 2D network layout was set by the Kamada-Kawai algorithm. The vertical positions reflect the community landscape values of the nodes on a linear scale. Nodes were colored as the module of their maximum membership. Panel B: centrality and bridgeness of yeast date- and party-hubs. Hubs having more than 8 neighbors and non-hubs with less neighbors were positioned on the scattergram according to their ModuLand centrality (x-axis, the height of the community landscape) and ModuLand bridgeness (y-axis) as defined in Section V.6.d. in the Electronic Supplementary Material S1. Date- and party-hubs are marked with red circles and blue triangles, respectively, while non-hub proteins are represented by gray crosses. The inset shows a double logarithmic plot of hubs with large centrality.

Discrimination of date- and party-hubs of protein interaction networks, i.e. proteins sequentially or simultaneously interacting with a large number of neighbors, is a rather difficult task [32]–[37]. We hypothesized, that among date-hubs and party-hubs of similar centrality, date-hubs may have a higher bridgeness (i.e. they are more overlapping between modules of the network). This assumption was substantiated by the inter-modular position of date-hubs [34], [36] and by the similarly high efficiency of bridgeness-based and date-hub-based network disintegration (cf. Figure 5 with Figure 2 of [34] and [37]). The identification of the overlapping modules of a high-confidence yeast protein-protein interaction network [25] resulted in a number of modules with well-known functions (Figure 6A and Figure S1.14 in the Electronic Supplementary Material S1). We calculated the bridgeness and centrality measures of the individual proteins, and plotted these values on Figure 6B. The separation of date- and party-hubs represented by the line of Figure 6B classified 84 party-hubs correctly of the total of 201, and 307 date-hubs of the total of 318. This result becomes even more convincing, if we consider that 10 out of 11 incorrectly identified date-hubs (91%) and 89 out of 117 incorrectly identified party-hubs (76%) have been potentially misclassified, if comparing them to the consensus of classifications [32]–[36]. In conclusion, by the help of the novel measures of the ModuLand-based analysis, we were able to discriminate between date- and party-hubs, thus predicting the dynamic behavior of network nodes using only the topological information of their network.

### General Characterization of the ModuLand Method Family

After the examples showing the utility of the ModuLand method family to determine overlapping modules of a variety of model and real world networks in this section we will summarize the characteristics of the ModuLand method family. In principle both the calculation of the influence functions and the determination of the community landscape hills are demanding problems, requiring specific solutions depending on the precise nature of the analyzed network. However, by constructing the community landscape, the small details of the influence functions get averaged out, therefore in practical cases fast and approximate solutions of the mentioned problems become possible and sufficient. This is the reason why rather simple influence function calculation methods (like the NodeLand algorithm) perform well on various kinds of real-world networks. On the other hand, the module membership value of any given node is obtained as the sum of the module membership value of the links of the given node, thus the small details of the hill determination step get also averaged out. The summation of the link module membership values provides an overlapping modularization of the nodes even in the absence of an overlapping modularization of the links themselves. (A similar situation is described in ref. [14].) To summarize, we divided the very challenging problem of module determination into two likewise hard subproblems, but fortunately in most cases a relatively fast, approximate treatment of these subproblems provided sufficiently fine modularizations in the end. However, depending on the precise nature of the application, it is possible, or even advised to devise a more elaborate treatment of the subproblems of influence function calculation and community landscape hill determination.

Several widely used efficient network modularization methods [2], [7] can be interpreted as parts of the ModuLand method family either by 1.) identifying the underlying influence function calculation method or by 2.) identifying the community landscape directly (Section IV.4. in the Electronic Supplementary Material S1).

#### Previous methods as potential influence function calculation methods of the ModuLand method family

As an important example for the first case, Bagrow and Bollt [38] define local communities by the spreading of *l*-shells from the nodes of the network, which are suitable as influence functions for the ModuLand method family. The recent work of Roswall and Bergstrom [5] published during the course of the current study [39] uses the probability flow of random walks to construct a map of scientific communication yielding non-overlapping modules. Pons and Latapy also use [11] random walks in their algorithm called ‘Walktrap’ to define a similarity value for merging communities. The random walks used in these methods can be interpreted also as influence functions. The method of Lancichinetti et al. [23] iteratively finds local modules optimizing a local fitness function. However, instead of executing the local module finding for each node of the network, it is only executed for nodes not contained in any local module yet. This local module finding step can be inserted into the ModuLand method family as a (binary) influence function calculation method. (Note that executing the influence function calculation method only for a fraction of nodes is a possible valid approximation method inside the ModuLand method family, too.) The method of Lancichinetti et al. [23] does not yield fine information about the membership strength of the nodes to different modules as the ModuLand method family does, but yields binary containment information instead. The method and the ModuLand method family have different approaches for the determination of hierarchical levels (see Section VII. in the Electronic Supplementary Material S1).

#### Previous methods as potential community landscape identification methods of the ModuLand method family

As examples for the second case, namely, for the direct identification of the community landscape in previous methods, we briefly summarize the previously described network landscapes. Previous network landscape construction methods used clustering coefficients [33], edge number per visualized network unit area [40], loop-coefficients [41], or degrees [42] to define the landscape-height. The “*leading eigenvector method*” of [15] is able to divide the network in two (or if applied recursively, more) non-overlapping communities maximizing the modularity measure Q. Both the ModuLand method family and modularity-based methods let their users adapt to the specificities of the analyzed network. However, in case of the ModuLand method family this adaptation is achieved by the choice of the sub-steps (like the community landscape construction method), while the Q modularity-based methods require a null-model to be chosen, which is reflecting the experimenter's expectations about the network. The “*community centrality*” introduced in [15], just as any centrality measure, is a valid basis of forming a ModuLand community landscape, therefore making it possible to include this modularity-based method into the ModuLand method family. Recently, a number of publications showed a ‘hidden metric space’ behind network topologies, which also links the network structure to a landscape-type representation [43]. Hinneburg and Keim [44] used the density function landscape to determine non-overlapping clusters for the traditional data clustering task, but did not calculate the overlaps based on the hill detection as defined in ModuLand method family. Actually none of the methods mentioned above and listed in Table S1.2 in the Electronic Supplementary Material S1 use the hills of the landscape to determine the modular structure. Evans and Lambiotte [45] show that meaningful modules can be found in networks by finding modules of links instead of nodes, so that nodes can trivially belong to multiple modules, if its links do. However, this method does not give the fine information about the membership strength of the nodes to different modules as can be uncovered with the ModuLand method family.

New modularization methods can easily be generated by taking an existing ModuLand modularization protocol, and changing any of its influence function calculation, community landscape generation, or hill determination methods. Additionally, former methods yielding non-overlapping modules (which can be interpreted as the application of the threshold-based hill determination method) can be upgraded to overlapping modularization methods using the local maxima-based module determination approach of the ModuLand method family (for details see Section IV.4. in the Electronic Supplementary Material S1).

Enriching the binary, yes/no module membership assignment of many previous methods, the ModuLand method family gives a continuous scale for the association of each link and node to all modules (Figure S1.7 in the Electronic Supplementary Material S1). To define the number of modules of a link or node the ‘effective number’ of modules was introduced (see Section V.6.b. in the Electronic Supplementary Material S1), which is a threshold-less, continuous measure based on the effective size of support of a probability distribution [46]. Additionally, the ModuLand method allowed the definition of further measures characterizing e.g. the centrality and bridgeness of network nodes and links (see Sections IV. and V.6. in the Electronic Supplementary Material S1).

### Selecting the Appropriate Method of the ModuLand Method Family

In the ModuLand approach we divided the very challenging problem of module determination into two likewise hard subproblems: the influence function determination (1); and the determination of hills of the resulted community landscape (2). Although in most cases a relatively fast, approximate treatment of these subproblems provides sufficiently fine modularizations in the end, in the following section we give a brief guide to select the optimal algorithms for these subproblems.

1. As we mentioned earlier, the determination of the influence functions requires a network-dependent perturbation-flow simulation on the network. However, we saw, that the details of the influence functions usually average out during the community landscape construction, which justifies the use of less specific, faster approximations. We prefer to use the LinkLand algorithm on undirected networks, which is found to provide an acceptable compromise between precision and speed. However, on directed networks we suggest to use the PerturLand algorithm (see Section IV.2. in the Electronic Supplementary Material S1) instead. We believe that our influence function calculation algorithms (NodeLand, LinkLand, and PerturLand) present only the first steps in the direction towards novel accurate and fast influence simulation techniques.
2. For the hill determination on the community landscape we presented two main approaches (for other possibilities see Section V. in the Electronic Supplementary Material S1): the centrality threshold-based hill determination and the local maxima based hill determination approaches. The centrality threshold-based hill determination approach is appropriate whenever the goal of the analysis is to find modules without overlapping regions. In order to determine the overlaps between the modules we suggest to use one of the local maxima based methods. While for practical purposes we suggest the use the ProportionalHill method, the most detailed module overlap information is acquirable with the computationally more expensive TotalHill method. If we need only the most important overlaps between the modules, we may use the GradientHill method, as described in Section V.2.b. in the Electronic Supplementary Material S1.

We note that although the local maxima-based approaches we described in this paper (including the ProportionalHill method suggested above) outperform the traditional threshold-based approach in terms of overcoming the giant-component problem and producing continuously overlapping modules, nevertheless they also have their own drawbacks. When applying the local maxima-based approach on a ‘noisy’ community landscape, each local maximum will result a new (and possibly highly overlapping) module. Therefore we routinely applied a simple, yet effective post-processing step for merging the groups of extremely overlapping modules (having a correlation higher than 90%) (see Section VI. in the Electronic Supplementary Material S1).

To summarize, the hill-finding approach, which is the second phase of the ModuLand methods, gives an additional layer of flexibility, where the relatively inaccurate results of simpler hill definitions, and the large computational costs of more accurate optimization processes can be tailored to the network and to the experimenter's needs and possibilities.

## Discussion

The ModuLand method family we introduced in this paper and in part in an earlier patent application [39] is a novel, integrative approach, which includes also the usual partitioning techniques such as the threshold-based hill determination methods over an appropriate community landscape. However by using local maxima based hill determination methods, it yields overlapping modules over any community landscape. Thus, our approach is suitable to extend the previous partitioning techniques to find overlapping modules. We presented novel, special examples for influence function calculation (NodeLand, LinkLand and PerturLand algorithms), which form the basis of the community landscape construction. Moreover, the identification of modules as hills of the community landscape is a new approach, including traditional threshold-based algorithms and the novel local maxima based algorithms (as our ProportionaHill, GradientHill and TotalHill methods). The Moduland method family defines link-based modules and results in pervasively overlapping modules as some of the few most recent approaches [14], [45] published well after our initial studies [39]. Previous methods using local community detection or yielding overlapping modules (Table S1.2 in the Electronic Supplementary Material S1) [4], [7] can be interpreted as special cases of our ModuLand method family, if appropriate hill determination techniques or community landscape construction methods are designed.

The extensive and rich overlaps, network hierarchy, as well as the novel centrality and bridgeness measures uncovered by the ModuLand method can be used for the identification of long-range, stabilizing weak links, for the determination of the recently described creative, trend-setting nodes governing network development and evolution [47], for prediction of missing links or nodes, for network classification and for the design of efficient information transfer to name only a few of the many possibilities. Module overlaps might play a key role in the disconnection and synchronization of modules of complex systems, and their re-assembly during and after crisis, respectively. We invite our colleagues to design novel versions of the framework we gave, and to explore the above and other examples.

## Materials and Methods

### Networks

#### Network science co-authorship network

The giant component of the undirected, un-weighted network science co-authorship network contained 379 nodes and 914 links [15].

#### Karate club social network

The weighted and undirected social network of a karate club has been reported by W. Zachary [19] containing 34 nodes and 78 links. As the members of the karate club have split into two factions later, the network became a gold-standard of module determination methods [1]–[5], [7].

#### Word association network

The giant component of Appendix A of the University of South Florida word association network (http://www.usf.edu/FreeAssociation/) [20] with removed link directions contained 10,167 nodes and 63,788 weighted links, where weight refers to the association strength (see Section I.3. in the Electronic Supplementary Material S1).

#### School friendship network

The giant component of the high school friendship Community-44 of the Add-Health database (http://www.cpc.unc.edu/projects/addhealth) [27] with removed link directions contained 1,127 nodes and 5,096 weighted links, where weights represent the strengths of friendships (see Section I.4. in the Electronic Supplementary Material S1).

#### Power-grid network

The un-weighted and undirected network of the USA Western Power Grid [31] contained 4,941 nodes and 6,594 links (http://vlado.fmf.uni-lj.si/pub/networks/data/map/USpowerGrid.net).

#### Yeast protein-protein interaction network

The giant component of the un-weighted and undirected yeast protein-protein interaction network [25] contained 2,444 nodes and 6,271 links, covering approximately half of the yeast genome and the most reliable (‘strongest’) ∼3% of the expected number of total links. All these network data are included in the ModuLand program package downloadable from our homepage: http://www.linkgroup.hu/modules.php.

## Supporting Information

Electronic Supplementary Material S1 In this Electronic Supplementary Material S1 we give a detailed description of the ModuLand network module determination method family. This integrative method is based on the construction of community landscapes from influence functions. In Section IV. we describe three versions of the influence function calculation algorithms, the NodeLand, LinkLand and PerturLand algorithms in detail. As the next step. the combination of influence functions to a community landscape is shown. We demonstrate the wide applicability of the ModuLand method to accommodate previous community detection methods in the examples of the BetweennessCentralityLand (BCLand) and CliqueLand community landscape determination methods resulting in distinct and overlapping network modules, respectively. In Section V. we show the local maxima-based identification of modules as hills of the community landscape. The module membership of network nodes and links is calculated using one of the developed module membership assignment methods, such as the GradientHill, ProportionalHill or TotalHill methods yielding modules of minimal, fair or detailed overlaps, respectively. In Sections VII. And VIII. we also show that the ModuLand method family enables a hierarchical analysis of network topology and the construction of a zoom-in network visualization method. Besides the detailed description of the ModuLand method the Electronic Supplementary Material S1 also contains 14 Supplementary Figures and their Supplementary Discussion, as well as a detailed summary of 18 module definitions, 129 different modularization methods, 13 module comparison methods as 5 Supplementary Tables and 396 references.
(4.88 MB PDF)
